# Changes in dietary habits during Covid-19 lockdown in Egypt: the Egyptian COVIDiet study

**DOI:** 10.1186/s12889-023-15777-7

**Published:** 2023-05-25

**Authors:** Khaled Abdelkawy, Fawzy Elbarbry, Soha M El-masry, Amr Y. Zakaria, Celia Rodríguez-Pérez, Noha M El-khodary

**Affiliations:** 1 Department of Clinical Pharmacy, Faculty of Pharmacy, Kafrelsheikh University, Kafr el-Sheikh, Egypt; 2 Pacific University School of Pharmacy, Oregon, USA; 3 Department of Pharmaceutics, Faculty of Pharmacy, Damanhour University, Damanhour, Egypt; 4 Department of Pharmacy Practice, Faculty of Pharmacy, Horus University, New Damietta, Egypt; 5 Department of Nutrition and Food Science, University of Granada, Campus Universitario de Cartuja, Granada, 18017 Spain; 6 Biomedical Research Centre, Institute of Nutrition and Food Technology (INYTA) ‘José Mataix’, University of Granada, Avenida del Conocimiento s/n, Granada, 18071 Spain; 7 Instituto de Investigación Biosanitaria ibs.GRANADA, Granada, 18012 Spain

**Keywords:** COVID-19, Diet, Egypt, Lockdown, Physical activity, Mediterranean diet

## Abstract

**Purpose:**

COVID-19 lockdown changed social habits and lifestyle, including dietary habits, of people worldwide. However, limited information is available about these changes in Egypt. This cross-sectional study investigates the effects of COVID-19 lockdown on dietary habits among the Egyptian populations.

**Methods:**

An online questionnaire, based on sociodemographic data and dietary adherence in accordance with the validated PREDIMED MedDiet Adherence Screener (MEDAS), was used all over the Egyptian governorates. The dietary changes were statistically evaluated for significance in relation to age, gender, body mass index (BMI), education level and governorates.

**Results:**

A total of 1010 participants (76% aged below 36 years, 77% female, 22% obese, and 62% university-level education) answered the questionnaire. Respondents ≤ 20 years had a significant increase in weight and consumption of carbonated beverages, commercial pastries, fried and fast food. Egyptians > 50 years old had a significant decrease in physical activity. Underweight people (less than 3% of participants) increased their fast food intake with a prominent rise in weight. However, obese people increased cooking frequency and increased eating times with a decrease in physical activity. Male participants reported increased intake of carbonated beverages and fast food, while female participants increased the intake of homemade pastries with a significant decrease in physical activity. Approximately 50% of participants with postgraduate education reported decreased intake of fast food and carbonated beverages as well as decreased body weight. Residents of Cairo showed a significant increase in vegetable intake, and fried food intake with a decrease in seafood consumption. Participants from the Delta region had a significant increase in pastries intake.

**Conclusion:**

The findings of this study explored the need for increasing awareness about healthy lifestyle in future lockdown periods.

**Supplementary Information:**

The online version contains supplementary material available at 10.1186/s12889-023-15777-7.

## Introduction

COVID-19 is a severe acute respiratory syndrome caused by SARS coronavirus 2 (SARS-CoV-2). It affects the lower respiratory tract causing pneumonia in humans [1]. Recent reports showed that the COVID-19 is primarily transmitted through respiratory droplets or contact routes either by close contact with another one who is carrying the virus [2] or through fomites in the immediate environment around the infected person [3]. On January 2020, and due to the high spreading rates of the virus in China and worldwide, the World Health Organization (WHO) Emergency Committee declared it as a global health emergency [4] and later on as pandemic disease with recommendation of total lockdown of the population in their homes [5].

In response to the rapid spread of infections, the Egyptian government gave strict decisions by ordering Egyptian populations to stay at home from March 15 till the end of June 2020, to prevent the spread of COVID-19. Additionally, the Egyptian government has taken crucial measures to combat the pandemic such as closing schools, kindergartens, religious places, airports, and malls, as well as preventing social gatherings. During the lockdown period, people were only allowed to buy their essentials from supermarkets and pharmacy stores with strict adherence to maintaining social distance.

Being unprecedented in the recent history of Egypt, such lockdown introduced major modifications in the population´s norm. Restriction of staying at home greatly affected social habits and life style such as cooking habits, watching TV, smoking, alcohol drinking, exercise, and sleeping habits [6–8]. During lockdown, it was difficult to get fresh groceries and shortages of certain food products happened nationwide. As recognized by The Food and Agriculture Organization (FAO), the COVID-19 pandemic disrupted food chains around the world, affecting both supply and demand [9].

In addition, staying at home affected physical activities like practicing exercise, and outdoors walking and in-gym physical activity [8]. Such changes in physical activities are expected to create sedentary behavior that is known to increase risk of morbidity and cardiovascular diseases (CVD) [6–8]. Furthermore, the decline in physical activity could affect energy requirement and food intake. Similarly, the lockdown period influenced psychological state causing boring feeling, fearing sensation, teleworking stress, and social isolation especially for the elderly [10]. These psychological and physical changes may predispose people to many disorders that require medical attention to control weight and psychological disorders with possibility of many drug interactions and medication errors [11].

Also, the COVID-19 pandemic altered healthy habits of populations by seeking for immune-boosters mainly from herbal products or seeking healthy foods that improve immunity especially for those with disability, immune-compromised, elderly, and people with higher CVD risk. At the time of writing this manuscript, there was no approved specific drug to treat or prevent COVID-19 infection. Therefore, following a healthy and well-balanced diet was important to enhance the body’s immune response. Gene expression levels of most of the inflammatory mediators of COVID-19 are influenced by food and can modulate the processes of inflammation and oxidative stress during COVID attack [12]. The WHO provided many nutritional advices during the lockdown [13].

Consequently, changes in dietary and behavioral habits during the COVID-19 lockdown were expected in Egypt. However, limited information regarding these changes in dietary habits is available during the COVID-19 lockdown period. Therefore, this study aimed to examine the effects of COVID-19 lockdown on dietary habits among the adult Egyptian populations using a validated questionnaires distributed among the Egyptian governorates to help the Egyptian Ministry of health formulate their recommendations and nutrition policies for population in case of future lockdown periods [14].

## Materials and methods

### Study design and participants

A cross-sectional study within the COVIDiet project [15]“clinical trials registration number NCT04449731” was conducted with Egyptian individuals who were encouraged to take part in the current study. The inclusion criteria were as follow: adults (≥ 18 years old) population while the exclusion criteria included neonates and people with comorbid conditions like thyroid, diabetes, ascites, active hepatic and renal disorders to ensure that changes are mainly due to COVID-19 lockdown. The questionnaire was completely voluntary and anonymous. The study was approved by the Research Ethics Committee of the University of Granada (1526/CEIH/2020) and it was performed following the Helsinki Declaration, and approved by the University of Kafrelsheikh’s Research Ethics Committee (KFS-Ph-003/20). Before beginning the questionnaire, participants were informed about the purpose of the study and requested permission to use and publish the data from the study. The questionnaire was launched during the lockdown period in Egypt between March-August 2020.

### Instruments and variables

The questionnaire (Supplementary Table S1) included many items divided into three main sections. The first one included socio-demographic information items such as sex, age, place of residence, dependent children, weight, height, and level of education. The second part of the questionnaire included the validated 14-items PREDIMED Mediterranean diet (MedDiet) Adherence Screener (MEDAS-14) with some modifications [16]. The questionnaire is based on a multiple choice close ended question that, together with the way of evaluation, have been previously described [17]. In brief, one point was scored when participants employed olive oil for cooking, consumed ≥ 4 olive oil tablespoons, white meat instead red meat, ≥ 2 serving of vegetables, ≥ 3 pieces of fruit, < 1 serving of red meat, ≥ 7 glasses of wine per week, ≥ 3 servings of legumes, fish/seafood, or nuts, and ≥ 2 dishes seasoned with sofrito, < 2 servings of non-homemade pastries and preference of white meat vs. red meat. However, in this case, wine intake was excluded due to low intake incidence in Egypt as alcohol is prohibited in Islamic countries. In addition, 35 in-house questions aimed at investigating changes in their general dietary habits during the lockdown such as the changes in the intake of olive oil, vegetables, fruits, red meats, butter, beverages, honey, legumes, fish, pastries, nuts, chicken, cooked food, pasta, rice, fried food, and fast food added to changes in physical activity and body weight of the participants. The internal validity (reliability) of the survey questions were piloted with 25 individuals who completed the questionnaire twice with 5 weeks in between and measured the reliability using the Cronbach’s alpha. The Egyptian COVIDiet study was coordinated by the Clinical Pharmacy Research Center, Faculty of Pharmacy Kafrelsheikh University. The Arabic translation can be obtained from the authors upon request.

### Procedure

To try to cover as much population as possible, the self-administered questionnaire was launched online and distributed using the Google Forms tool and it was broadcasted using instant messaging apps such as WhatsApp, social media such as Facebook and Twitter, social networking sites such as LinkedIn and ResearchGate through snowball sampling.

### Data and statistical analysis

Data analysis was carried out using Statistical Package of Social Sciences (SPSS) version 21 for Windows (SPSS, Inc., Chicago, IL, USA). The self-reported weight (in kilograms) and height (in meters) were used to calculate BMI from this formula ($BMI=\frac{Weight \left(kg\right)}{Height \left({m}^{2}\right)}$). Sociodemographic characteristics, weight status, BMI, and changes in dietary behaviors were described, when applicable, by frequency, percentage, and mean +/- standard deviation. Student’s t-test (continuous normal) and Chi square test (for categorical data) were used to analyze differences in means or proportions of the tested variables. Cronbach’s alpha was used to test the reliability (internal consistency) of the scales of the questionnaire. A p-value of less than 0.05 (*p* < 0.05) was considered statistically significant.

## Results

A total of 1010 responders were included in the study; of which 77.3% were females, 22.7% were males, 8.6% aged ≤ 20, 67.6% aged 21–35, 21.7% aged 36–50, and 2% aged > 50. Approximately 62% of the participants had university education, 31% had postgraduate education and the remaining had only secondary education as a terminal degree. According to their calculated body mass index (BMI), 39% of the respondents were overweight while 22% were obese. Nearly half of the subjects resided in Alexandria, while 34% in the delta region, and 11% in Cairo. The socio-demographic and anthropometric characteristics of the study participants are reported in Table 1.

**Table 1:** Demographic and anthropometric characteristics of questionnaire respondents during the COVID-19 lockdown in Egypt

Variable	Frequency, n (%)
**Age (Years old)**	
≤ 20	87 (8.6)
21–35	683 (67.6)
36–50	219 (21.7)
> 50	21 (2.1)
**BMI (kg/m** ^**2**^ **)**	
< 18.5	23 (2.3)
18.5-< 25	369 (36.5)
25-< 30	394 (39.0)
> 30	224 (22.2)
**Gender**	
Male	229 (22.7)
Female	781 (77.3)
**Children in Care**	
Yes	469 (46.4)
No	541 (53.6)
**Place of Residence**	
Family home	877 (86.8)
Alone	133 (13.2)
**Educational Level**	
University	629 (62.3)
Postgraduate	315 (31.2)
Secondary	66 (6.5)
**Governorate**	
Alexandria	504 (49.9)
Cairo	342 (33.9)
Delta	114 (11.3)
Others	50 (4.9)

**Fig. 1:**
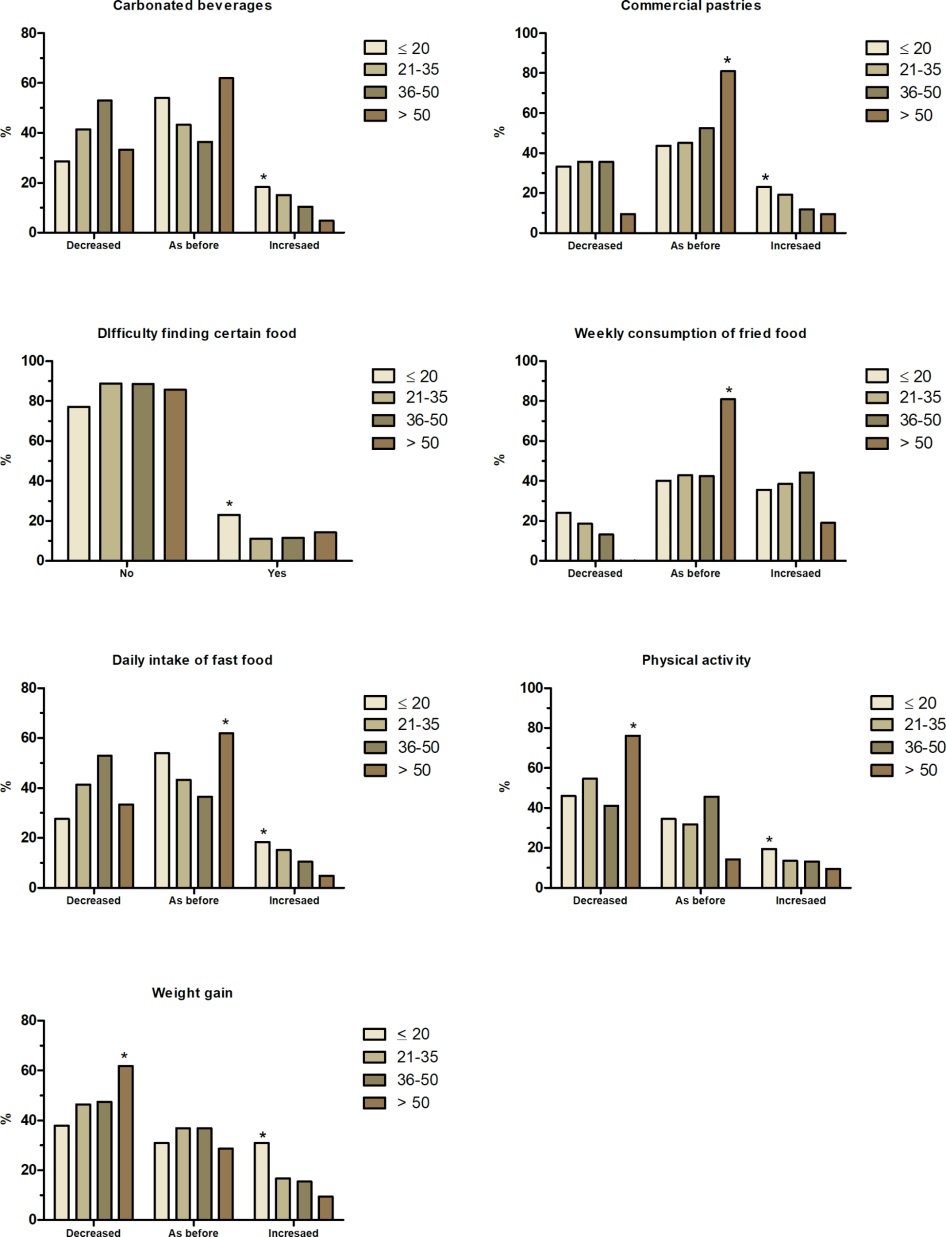
Dietary and lifestyle modifications by age during the COVID-19 lockdown in Egypt. * Indicates significant difference at p < 0.05

The implemented survey questions showed good internal validity with Cronbach’s alpha = 0.83. Our study identified significant changes related to lockdown among the different age groups, especially with the daily intake of carbonated and sugary beverages, honey, commercial pastries, nuts, fried food, and fast food. In addition, there was a significant change in meals frequency (breakfast, lunch, or dinner) outside home and a difficulty to find certain types of food during the COVID-19 lockdown. Furthermore, the lockdown caused significant changes in physical activity and weight gain. As illustrated in Fig. 1, people ≤ 20-year-old significantly increased the intake of carbonated beverages (18.4%), commercial pastries (23%), fried food (36%), and fast food (17%). Moreover, this age group demonstrated a significant rise in body weight (31%), significant increase in physical activity (19.5%) and difficulties to find certain food (23%) like honey, olive oil, broccoli, orange and pineapple. A significant portion (> 60–80%) of respondents > 50-year-old reported no change in consumption of commercial pastries, fried food, and fast food during the lockdown. Despite a reported significant reduction (> 78%) in physical activity, more than 60% of respondents > 50-year-old reported decrease in body weight during the lockdown. Approximately 40% of participants aged 21–50 reported no changes in the consumption of carbonated and sugary beverages, commercial pastries, fried food and fast food, and weight gain during the lockdown.

Table 2 details the baseline characteristics of questionnaire respondents by age. Except for the place of residence (family home versus alone) and BMI, all age groups demonstrated statistically significant differences (*p* < 0.05) in all tested variables.

**Table 2:** The baseline characteristics of questionnaire respondents by age

	Age (Years)					
	Total (n = 1010)	≤ 20 (n = 87)	21–35 (n = 683)	36–50 (n = 219)	> 51 (n = 21)	*p-Value* ^*1*^
**Place of residence**						
Family home	877 (86.8%)	82 (94.2%)	596 (87.3%)	182 (83.1%)	17 (80.9%)	0.055
Alone	133 (13.2%)	5 (5.7%)	87 (12.7%)	37 (16.9%)	4 (19.0%)	
**Gender**						
Male	229 (22.7%)	22 (25.3%)	148 (21.7%)	48 (21.9%)	11 (52.4%)	0.010*
Female	781 (77.3%)	65 (74.7%)	535 (78.3%)	171 (78.1%)	10 (47.6%)	
**Children in Care**						
No	541 (53.6%)	85 (97.7%)	416 (60.9%)	29 (13.2%)	11 (52.4%)	< 0.001*
Yes	469 (46.4%)	2 (2.3%)	267 (39.1%)	190 (86.8%)	10 (47.6%)	
**Educational Level**						
University	629 (62.3%)	52 (59.8%)	467 (68.4%)	96 (43.8%)	14 (66.7%)	< 0.001*
Postgraduate	315 (31.2%)	1 (1.1%)	194 (28.4%)	115 (52.5%)	5 (23.8%)	
Secondary	66 (6.5%)	34 (39.1%)	22 (3.2%)	8 (3.7%)	2 (9.5%)	
**Governorate**						
Alexandria	504 (49.9%)	47 (54.0%)	362 (53.0%)	84 (38.4%)	11 (52.4%)	< 0.001*
Delta	342 (33.9%)	40 (46.0%)	228 (33.4%)	72 (32.9%)	2 (9.5%)	
Cairo	114 (11.3%)	0 (00.00%)	65 (9.5%)	45 (20.5%)	4 (19.1%)	
Others	50 (4.9%)	0 (00.00%)	28 (4.1%)	18 (8.2%)	4 (19.0%)	
**BMI (kg/m** ^**2**^ **)**						
< 19	23 (2.28%)	6 (6.90%)	16 (2.34%)	1 (0.46%)	0 (00.00%)	0.25
19-24.9	369 (36.53%)	53 (60.92%)	268 (39.24%)	43 (19.63%)	5 (23.81%)	
25-29.9	394 (39.01%)	21 (24.14%)	268 (39.24%)	97 (35.62%)	8 (38.10%)	
> 30	224 (22.18%)	7 (8.05%)	131 (19.18%)	78 (35.62%)	8 (38.10%)	

Chi square test (for categorical data) and Student’s t-test (continuous normal data) were used to analyze differences in means or proportions of the tested variables. ^*1*^ Significant difference (*) was considered at p < 0.05

**Fig. 2:**
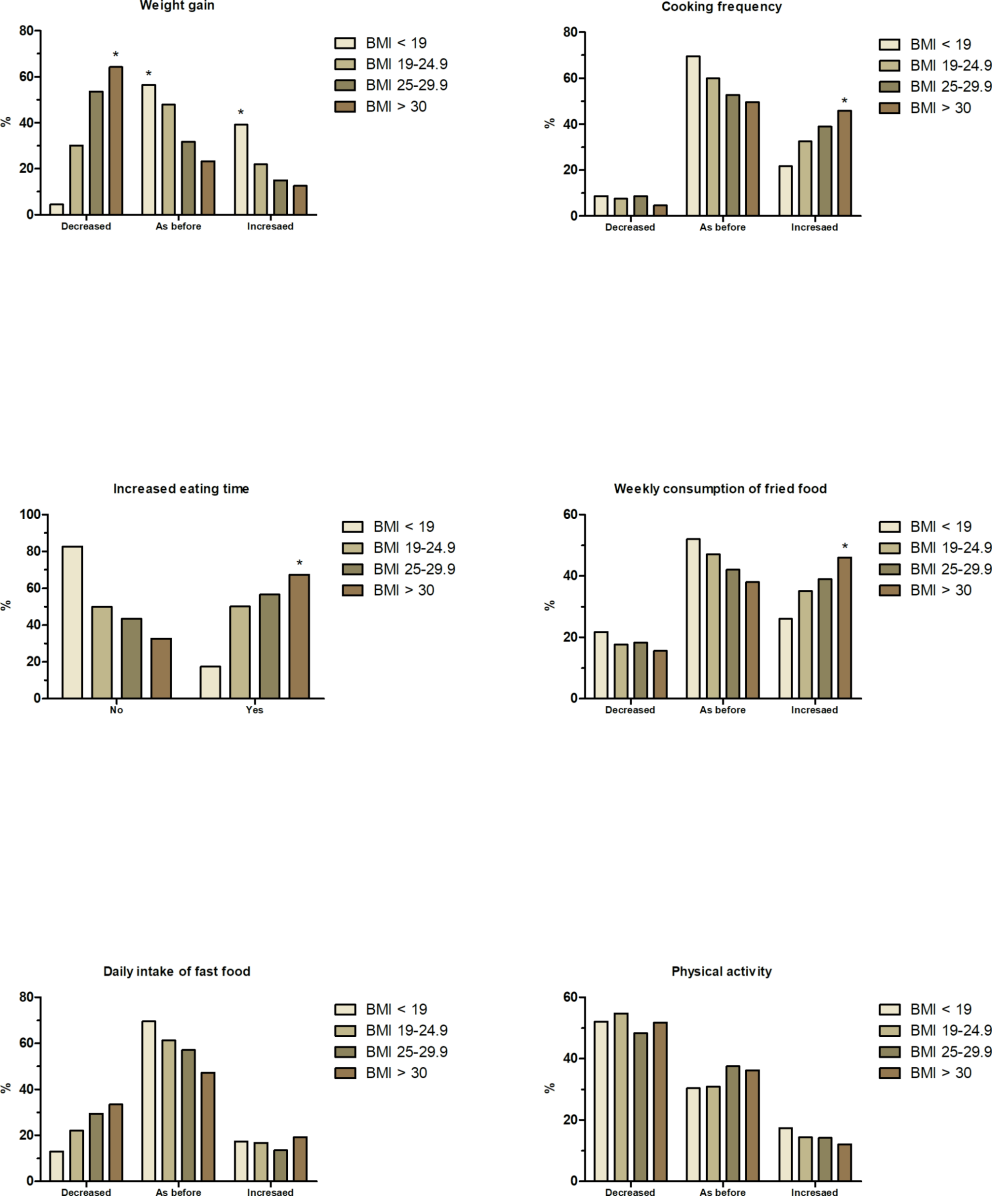
Dietary and lifestyle modifications by Body Mass Index (BMI) during the COVID-19 lockdown in Egypt. * Indicates significant difference at p < 0.05

The changes in participants’ body weight were reported in the form of BMI, calculated as kg/m^2^. According to the definition of WHO, BMI was categorized as underweight (BMI < 19; n = 23), normal weight (BMI = 19-24.9; n = 369), overweight (BMI = 25-29.9; n = 394), and obese (BMI > 30; n = 224). With relation to BMI, there were significant changes in daily intake of red meats, hamburgers, sausages or deli meats, butter, margarine and cream. In addition, there was significant changes in daily frequency of having meals (breakfast, lunch or dinner) outside of home and frequency of cooking compared to before the lockdown started. Furthermore, there was significant changes in the intake of fast food, weekly consumption of fried food, and eating times during the lockdown. Besides, there were both modifications of physical activity and weight gain during lockdown. Figure 2 shows the most notable and significant changes related to participants’ BMI during the lockdown. As shown from the figure, underweight people decreased the cooking frequency (9%) and increased the fast food intake (17.5%) with prominent rise in weight (39%) during lockdown. In contrast, obese people increased cooking frequency (46%) and increased eating time (67.5%) with prominent decrease in physical activity (22%), and minimal increase in body weight (12.5%) during lockdown. At least 50% of participants who have normal body weight reported no weight gain or changes in physical activity or dietary habits.

Table 3 details the baseline characteristics of questionnaire respondents by BMI. Except for the place of residence (family home versus alone) and gender, all BMI categories demonstrated statistically significant differences (*p* < 0.05) in all tested variables.

**Table 3:** The baseline characteristics of questionnaire respondents by BMI

	BMI (kg/m^2^)					
	Total (n = 1010)	< 19 (n = 23)	19-24.9 (n = 369)	25-29.9 (n = 3949)	> 30 (n = 224)	*p-Value* ^*1*^
**Place of residence**						
Family home	877 (86.8%)	20 (87.0%)	328 (89.0%)	343 (87.0%)	186 (83.0%)	0.24
Alone	133 (13.2%)	3 (13.0%)	41 (11.0%)	51 (13.0%)	38 (17.0%)	
**Gender**						
Male	229 (22.7%)	5 (21.7%)	81 (22.0%)	97 (24.6%)	46 (20.5%)	0.67
Female	781 (77.3%)	18 (78.3%)	288 (78.0%)	297 (75.4%)	178 (79.5%)	
**Children in Care**						
No	541 (53.6%)	19 (82.6%)	269 (72.9%)	194 (49.2%)	59 (26.3%)	< 0.001*
Yes	469 (46.4%)	4 (17.4%)	100 (27.1%)	200 (50.8%)	165 (73.7%)	
**Educational Level**						
University	629 (62.3%)	18 (78.3%)	241 (65.3%)	249 (63.2%)	121 (54.0%)	< 0.001*
Postgraduate	315 (31.2%)	3 (13.0%)	92 (24.9%)	126 (32.0%)	94 (42.0%)	
Secondary	66 (6.5%)	2 (8.7%)	36 (9.8%)	19 (4.8%)	9 (4.0%)	
**Governorate**						
Alexandria	504 (49.9%)	11 (47.8%)	180 (48.8%)	207 (52.5%)	106 (47.3%)	< 0.001*
Delta	342 (33.9%)	10 (43.5%)	144 (39.0%)	121 (30.7%)	67 (29.9%)	
Cairo	114 (11.3%)	2 (8.7%)	32 (8.7%)	47 (11.9%)	33 (14.7%)	
Others	50 (4.9%)	0 (0.00%)	13 (3.5%)	19 (4.8%)	18 (8.1%)	
**Age (years)**						
< 20	87 (8.6%)	6 (26.1%)	53 (14.4%)	21 (5.3%)	7 (3.1%)	0.025*
21–35	683 (67.6%)	16 (69.6%)	268 (72.6%)	268 (68.0%)	131 (58.5%)	
36–50	219 (21.7%)	1 (4.3%)	43 (11.6%)	97 (24.6%)	78 (34.8%)	
> 50	21 (2.1%)	0 (0.0%)	5 (1.4%)	8 (2.1%)	8 (3.6%)	

Chi square test (for categorical data) and Student’s t-test (continuous normal data) were used to analyze differences in means or proportions of the tested variables. ^*1*^ Significant difference (*) was considered at *p* < 0.05

**Fig. 3:**
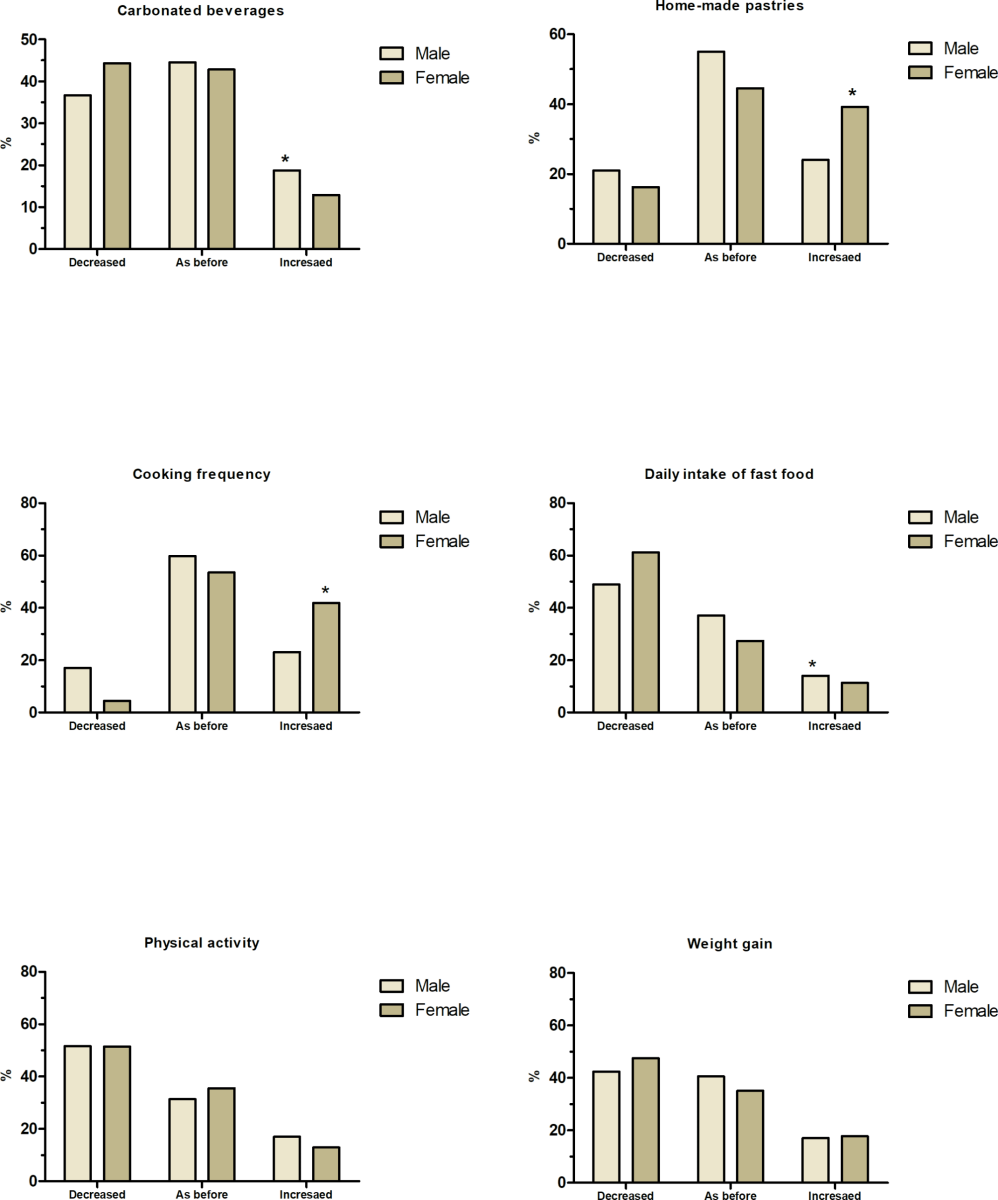
Dietary and lifestyle modifications by gender during the COVID-19 lockdown in Egypt. * Indicates significant difference at p < 0.05

The study also found significant modifications in food habits, physical activity, and weight changes in relation to gender during the lockdown. There were significant changes in daily intake of vegetables, butter, margarine or cream, honey, fish-seafood, cooked vegetables, pasta, rice, and frequency of meals outside of home between male and female participants. Besides, there were significant increase in carbonated beverages, homemade pastries, fast food and frequency of cooking during the lockdown, added to a significant change in physical activity. Figure 3 revealed the significant changes related to the lockdown, where male Egyptians increased the intake of carbonated beverages (21%), and fast food (14%). Female Egyptians, on the other hand, increased the intake of homemade pastries (39%), and cooking frequency (42%). Approximately 40–50% of the participants reported a decline in their physical activity and body weight regardless of the gender, without any statistically significant differences.

Table 4 details the baseline characteristics of questionnaire respondents by gender. Except for the place of residence (family home versus alone) and geographical location (governorates), both gender categories demonstrated statistically significant differences (*p* < 0.05) in all tested variables.

**Table 4:** The baseline characteristics of questionnaire respondents by gender

	Gender			
	Total (n = 1010)	Male (n = 229)	Female (n = 781)	*p-Value* ^*1*^
**Place of residence**				
Family home	877 (86.8%)	201 (87.8%)	676 (86.6%)	0.47
Alone	133 (13.2%)	28 (12.2%)	105 (13.4%)	
**BMI**				
≤ 19	23 (2.28%)	5 (2.2%)	18 (2.3%)	0.02*
19-24.9	369 (36.53%)	81 (35.3%)	288 (36.9%)	
25-29.9	394 (39.01%)	97 (42.5%)	297 (38%)	
> 30	224 (22.18%)	46 (20%)	178 (22.8%)	
**Children in Care**				
No	541 (53.6%)	153 (66.8%)	388 (49.7%)	< 0.001*
Yes	469 (46.4%)	76 (33.2%)	393 (50.3%)	
**Educational Level**				
University	629 (62.3%)	166 (72.5%)	463 (59.3%)	< 0.001*
Postgraduate	315 (31.2%)	44 (19.2%)	271 (34.7%)	
Secondary	66 (6.5%)	19 (8.3%)	47 (6%)	
**Governorate**				
Alexandria	504 (49.9%)	11 (47.8%)	180 (48.8%)	0.09
Delta	342 (33.9%)	10 (43.5%)	144 (39.0%)	
Cairo	114 (11.3%)	2 (8.7%)	32 (8.7%)	
Others	50 (4.9%)	0 (0.00%)	13 (3.5%)	
**Age (years)**				
≤ 20	87 (8.6%)	22 (9.6%)	65 (8.3%)	0.01*
21–35	683 (67.6%)	148 (64.6%)	535 (68.5%)	
36–50	219 (21.7%)	48 (21%)	171 (21.9%)	
> 50	21 (2.1%)	11 (4.8%)	10 (1.3%)	

Chi square test (for categorical data) and Student’s t-test (continuous normal data) were used to analyze differences in means or proportions of the tested variables. ^*1*^ Significant difference (*) was considered at p < 0.05

**Fig. 4:**
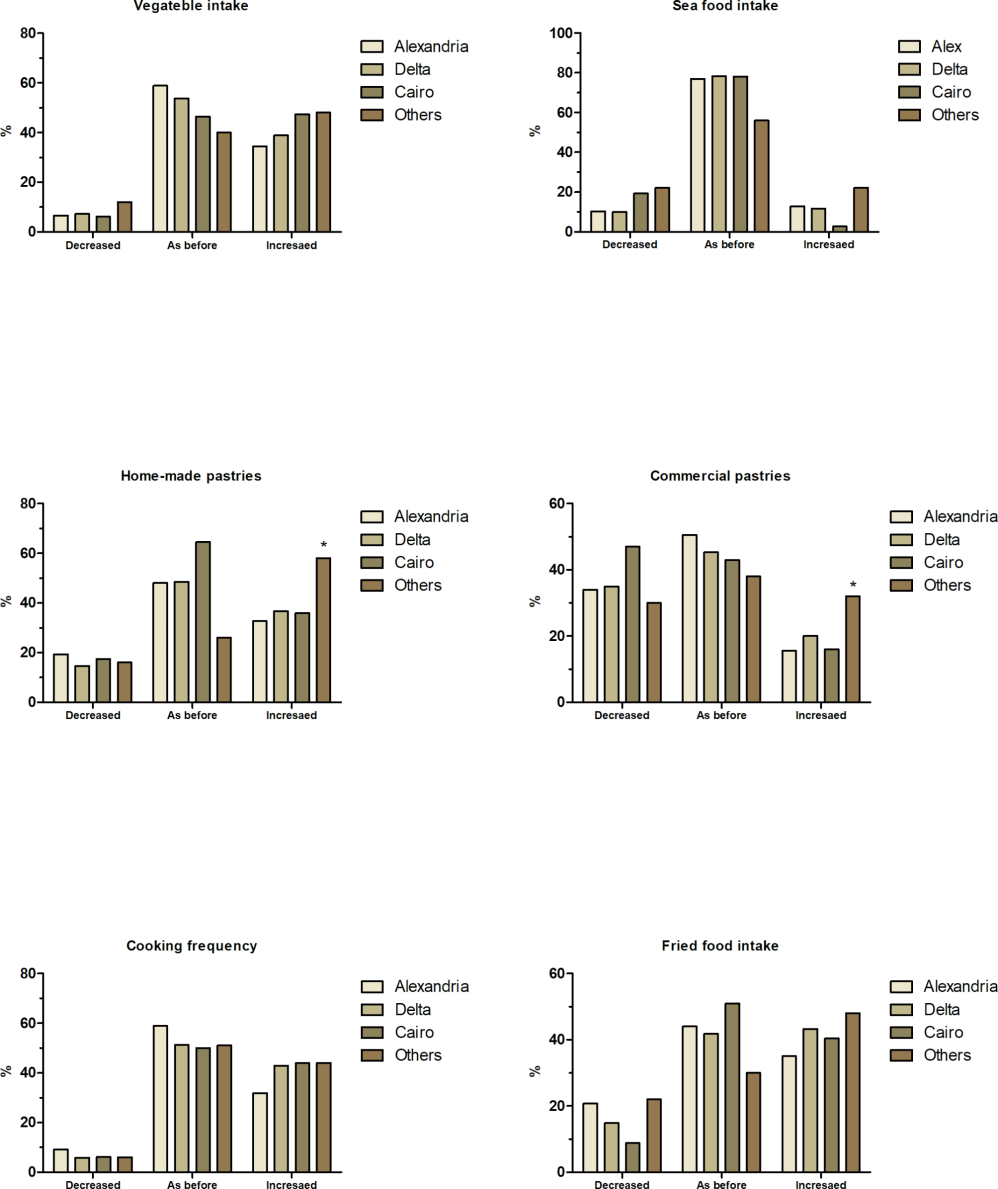
Dietary and lifestyle modifications by geographical location during the COVID-19 lockdown in Egypt. * Indicates significant difference at p < 0.05

Participants’ dietary habits and physical activity during lockdown also showed changes in relation to their geographical location, which was categorized into four main governorates: Alexandria (n = 504), Delta (n = 342), Cairo (n = 114), and others (n = 50). There were notable differences among the four governorates in the intake of olive oil, nuts, vegetables, fish-seafood, commercial (non-homemade) pastries, homemade pastries and fried-food during the lockdown with increasing in frequency of cooking. Figure 4 displays the significant changes during lockdown among governorates. Residents of Cairo showed an increase in vegetable intake (47%), fried food intake (40.5%) and cooking frequency (44%) with 19.5% and 41% reported a decrease in the intake of seafood and commercial pastries, respectively. Residents of the Delta governorates reported an increase in intake of commercial pastries (20%), homemade pastries (37%), and fired food (43.5%) with increasing cooking frequency in 43% of the respondents. However, participants from Alexandria showed a decrease in intake of commercial pastries (34%), homemade pastries (19.5%), fried food (21%) and cooking frequency (9%) during lockdown. Approximately 50% of participants from all governates reported weight decrease while 33–40% reported no changes in body weight. Statistical analysis did not reveal any significant differences among governorates, except for participants from “other governorates” who reported significant increase in consumption of pastries (commercial and home-made).

Table 5 details the baseline characteristics of questionnaire respondents by their geographical locations. Except for the place of residence (family home versus alone) and gender, all governorates’ categories demonstrated statistically significant differences (*p* < 0.05) in all tested variables.

**Table 5:** The baseline characteristics of questionnaire respondents by geographical location

	Governorates					
	Total (n = 1010)	Alex (n = 504)	Delta (n = 342)	Cairo (n = 114)	Others (n = 50)	*p-Value* ^*1*^
**Place of residence**						
Family home	877 (86.8%)	443 (87.9%)	301 (88.0%)	95 (83.3%)	38 (76.0%)	0.06
Alone	133 (13.2%)	61 (12.1%)	41 (12.0%)	19 (16.7%)	12 (24.0%)	
**Gender**						
Male	229 (22.7%)	123 (24.4%)	62 (18.1%)	31 (27.2%)	13 (26.0%)	0.09
Female	781 (77.3%)	381 (75.6%)	280 (81.9%)	83 (72.8%)	37 (74.0%)	
**Children in Care**						
No	541 (53.6%)	296 (58.7%)	198 (57.9%)	40 (35.1%)	7 (14.0%)	< 0.001*
Yes	469 (46.4%)	208 (41.3%)	144 (42.1%)	74 (64.9%)	43 (86.0%)	
**Educational Level**						
University	629 (62.3%)	329 (65.3%)	208 (60.8%)	64 (56.1%)	28 (56.0%)	0.045*
Postgraduate	315 (31.2%)	148 (29.4%)	102 (29.8%)	45 (39.5%)	20 (56.0%)	
Secondary	66 (6.5%)	27 (5.3%)	32 (9.4%)	5 (4.4%)	2 (4.0%)	
**BMI (kg/m** ^**2**^ **)**						
< 19	23 (2.3%)	11 (2.2%)	10 (2.9%)	2 (1.7%)	0 (00.0%)	0.029*
19-24.9	369 (36.5%)	180 (35.7%)	144 (42.1%)	32 (28.1%)	13 (26.0%)	
25-29.9	394 (39.0%)	207 (41.1%)	121 (35.4%)	47 (41.2%)	19 (38.0%)	
> 30	224 (22.2%)	106 (21.0%)	67 (19.6%)	33 (29.0%)	18 (36.0%)	
**Age (years)**						
≤ 20	87 (8.6%)	47 (9.3%)	40 (11.7%)	0 (00.0%)	0 (00.0%)	< 0.001*
21–35	683 (67.6%)	362 (71.8%)	228 (66.7%)	65 (57.0%)	28 (56.0%)	
36–50	219 (21.7%)	84 (16.7%)	72 (21.0%)	45 (39.5%)	18 (36.0%)	
> 50	21 (2.1%)	11 (2.2%)	2 (0.6%)	4 (3.5%)	4 (8.0%)	

Chi square test (for categorical data) and Student’s t-test (continuous normal data) were used to analyze differences in means or proportions of the tested variables. ^*1*^ Significant difference (*) was considered at p < 0.05

**Fig. 5:**
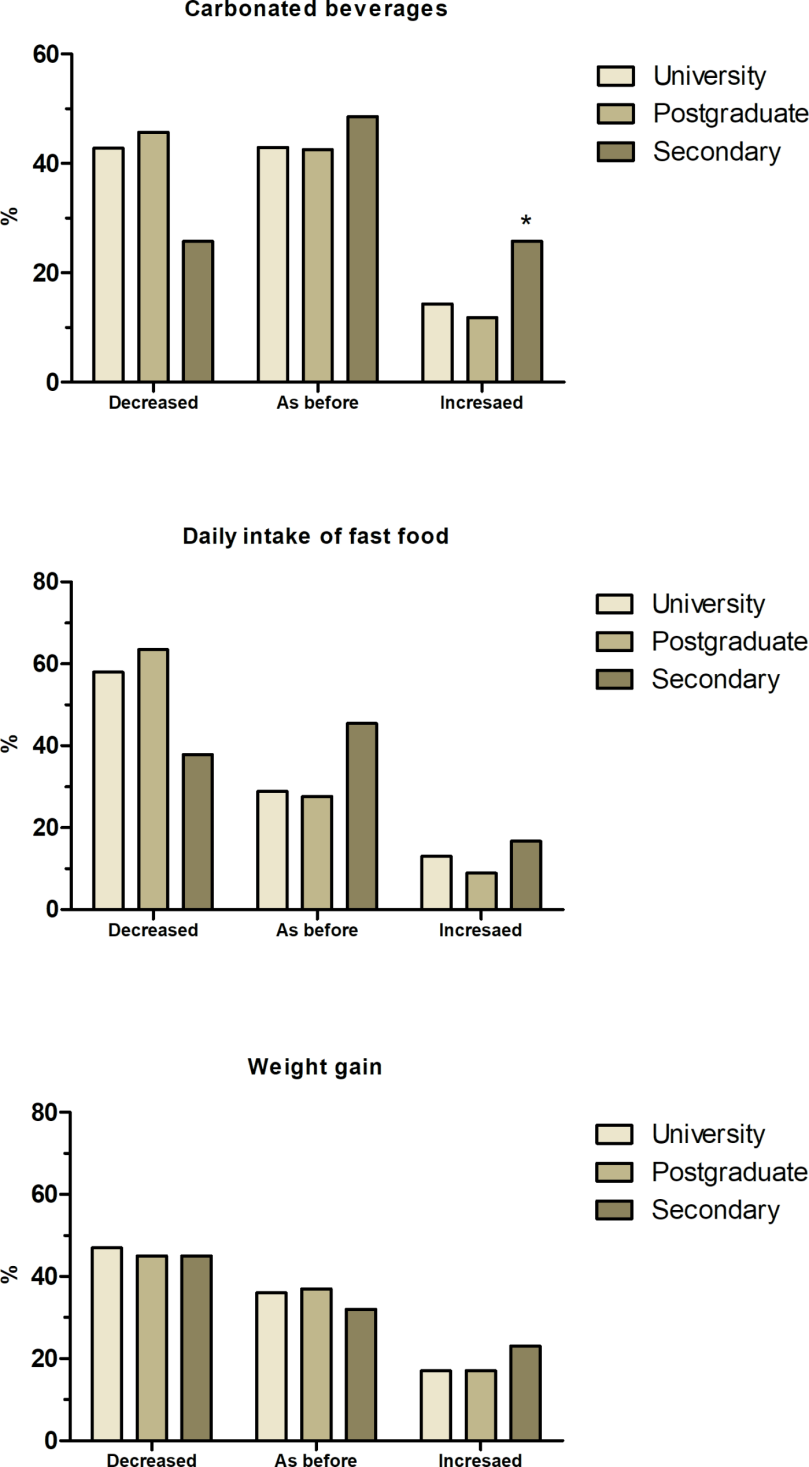
Dietary and lifestyle modifications by level of education during the COVID-19 lockdown in Egypt. * Indicates significant difference at p < 0.05

Similarly, our study revealed that educational level of participants demonstrated a significant effect on the food habits, physical activity, and weight changes during lockdown. Educational levels were categorized into university (n = 629), Postgraduate (n = 315), and Secondary (n = 66). There were significant differences in the intake of vegetables, honey, fish-seafood, cooked vegetables, pasta, rice, fried food among different educational level. Besides, during the lockdown, there were significant changes in the intake of carbonated beverages and fast food. Figure 5 shows the main changes related to the lockdown. Participants with secondary education showed increased intake of carbonated beverages (29%). Approximately 45% of all participants regardless of their education level reported a decrease in body weight, and 33% reported no change in their body weight during the lockdown.

Table 6 demonstrates the baseline characteristics of questionnaire respondents by their educational level. Except for the place of residence (family home versus alone), all educational background categories demonstrated statistically significant differences (*p* < 0.05) in all tested covariates.

**Table 6:** The baseline characteristics of questionnaire respondents by educational level

	Educational Level				
	Total (n = 1010)	University (n = 629)	Postgraduate (n = 315)	Secondary (n = 66)	*p-Value* ^*1*^
**Place of residence**					
Family home	541 (53.6%)	389 (61.8%)	98 (31.1%)	54 (81.8%)	0.07
Alone	469 (46.4%)	240 (38.2%)	217 (68.9%)	12 (18.2%)	
**Gender**					
Male	229 (22.7%)	166 (26.4%)	44 (14.0%)	19 (28.8%)	< 0.001*
Female	781 (77.3%)	463 (73.6%)	271 (86.0%)	47 (71.2%)	
**Children in Care**					
No	541 (53.6%)	389 (61.8%)	98 (31.1%)	54 (81.8%)	< 0.001*
Yes	469 (46.4%)	240 (38.2%)	217 (68.9%)	12 (18.2%)	
**Governorates**					
Alexandria	504 (49.9%)	329 (52.3%)	148 (47.0%)	27 (40.9%)	0.045
Delta	342 (33.9%)	208 (33.1%)	102 (32.4%)	32 (48.5%)	
Cairo	114 (11.2%)	64 (10.2%)	45 (14.3%)	5 (7.6%)	
Others	50 (05.0%)	28 (4.4%)	20 (6.3%)	2 (3.0%)	
**BMI (kg/m** ^**2**^ **)**					
< 19	23 (2.3%)	18 (2.9%)	3 (1.0%)	2 (3.0%)	< 0.001*
19-24.9	369 (36.5%)	241 (38.3%)	92 (29.2%)	36 (54.5%)	
25-29.9	394 (39.0%)	249 (39.6%)	126 (40.0%)	19 (28.8%)	
> 30	224 (22.2%)	121 (19.2%)	94 (29.8%)	9 (13.7%)	
**Age (years)**					
< 20	87 (8.6%)	52 (8.3%)	1 (0.3%)	34 (51.5%)	< 0.001*
21–35	683 (67.6%)	467 (74.2%)	194 (61.6%)	22 (33.3%)	
36–50	219 (21.7%)	96 (15.3%)	115 (36.5%)	8 (12.1%)	
> 50	21 (2.1%)	14 (2.2%)	5 (1.6%)	2 (3.1%)	

Chi square test (for categorical data) and Student’s t-test (continuous normal data) were used to analyze differences in means or proportions of the tested variables. ^*1*^ Significant difference (*) was considered at p < 0.05

## Discussion

The COVID-19 outbreak and subsequent lockdown has resulted in significant changes in the nutritional and activity patterns worldwide [6–8, 18]. These behavioral changes have been confirmed in several previous epidemiological studies [19–24]. For example, the cross-sectional questionnaire of Christopher Papandreou et al. on 1,002 adult participants in Spain and 839 in Greece during the year 2020 revealed interesting findings of lower restraint and external eating during the lockdown period [25]. Similarly, the cross-sectional questionnaire of Alomari et al. in 1844 Jordanian adults during the lockdown period between April and May of 2020, demonstrated significant changes in physical activity and sedentary behavior [26]. Changes like reduced physical activity and minimal intake of healthy food could result in negative health consequences. Therefore supportive healthy behaviors should be a public health priority.

The current findings revealed that Egyptians younger than 20 demonstrated increased body weight during the COVID-19 lockdown which could be associated with a rise of fast food, fried food, carbonated beverage, and commercial pastries intake during confinement. These changes might predispose this age category to more health complications like CVD, diabetes, hyperlipidemia, and obesity. Our findings are generally in line with a recent longitudinal study by Pietrobelli et al. who revealed the loss of weight control in obese children and adolescents in Verona, Italy during the lockdown [27]. Similarly, other studies reported increased consumption of cereals and grains (rice, noodles, pasta, and bread) by young adults during the pandemic lockdown and was associated with weight gai. [15, 28]. A Malaysian study revealed that more than 50% of the online learning undergraduates skipped meal (mostly breakfast) and 94% of participants snacked between meals [29]. Although this later study did not investigate the impact of the meal skipping on weight, it is believed that breakfast skipping can in fact cause more intake of energy dense food during the day, and potentially more weight gain. A possible reason behind this observation is the lack of stress management among this age group. Several systematic reviews have reported increased mental health problem and stress among teens and young adults during strict quarantine and lockdown conditions [30]. Perceived stress has been positively associated with unhealthy dietary behaviors and reduced physical activity [31]. Governmental agencies should prioritize establishing social and mentorship programs to help young adults to cope with stress and improve their dietary choices during lockdown.

Furthermore, during the lockdown period, participants older than 50 years had a significant decrease in consumption of commercial pastries, fast food and carbonated beverages. Moreover, they had a significant decrease in physical activity with no increase in body weight. The systemic review of Murillo Rezende Oliveira et al. revealed close findings to our study. The elderly population’s physical activity levels were decreased in the quarantine period of COVID-19 worldwide with negative impacts on their health [32]. Similarly, other studies suggested pandemic-induced psychological distress and sedentarism [33–36]. The WHO recommends at least 2.5 h of weekly moderate physical activity [37]. Therefore, reduced exercise may predispose senior citizens to many health and psychological problems. The findings of our study, and others, strongly suggest establishing educational programs about the need for regular exercise to help overcome potential health problem from the decrease of physical activity.

Our study has also demonstrated interesting influence of BMI on dietary and personal behavior of Egyptians during the COVID-19 lockdown. Approximately 74% of participants who are currently taking care of kids have shown higher percentage of obesity (BMI > 30). This might be related to a lack of self-care and increased level of stress especially when there is absence of social and economic support [38]. Our data shows a trend of increasing cooking frequency, eating time, consumption of fried and fast foods and reduction in physical activity with increasing body weight. Surprisingly, more than 60% of obese respondents reported a reduction in their body weight during the lockdown. This contradiction between unhealthy dietary/physical behavior and reduction in body weight requires further investigation. The perception that participants could have about the food portions could underestimate/overestimate their real intake. Also, overweight and obese respondents are more likely to underestimate their weight. In a similar study conducted to examine the perceived changes to a range of weight-related behaviors in British adults during social lockdown, participants with higher BMI reported lower levels of physical activity and diet quality, and an increased frequency of overeating [39]. There is an urgent need to counteract the environmental factors that predispose population to weight gain. Changing energy balance by 100 kilocalories per day through a combination of reducing energy intake and increasing physical activity could avoid weight gain which could be especially relevant during the lockdown periods. Having a clear behavioral target for weight gain prevention may be critical to halting the obesity during the pandemic [40].

The current study shed the light on the difference in physical and dietary behaviors among Egyptians with respect to their educational background. It was not surprising to have no participants with only primary education as this population is less likely to rely on and use smart technologies, which could have been a hindrance to participate in the study. Our study shows an increased consumption of carbonated beverages and fast food, and increased weight gain among respondents with secondary education compared to other populations. This might be related to the spread of junk-fast food chains around Egyptian schools as consistent with the study of Al-Otaibi et al [41]. Individuals with secondary education have lower social status and are less financially stable with expectation of low healthy dietary pattern compared to populations with higher educational background. It should be mentioned that our sample has only 66 participants with secondary education which represents only 6.5% of the whole study population. Further investigation is required to analyze these findings and evaluate the impact of educational background on dietary and physical patterns. On the other hand, approximately 40–60% of the participants with university or postgraduate education reported reduced consumption of fast food and carbonated beverages as well as lower weight gain during the lockdown. This might be attributed to their better choices in choosing healthy life style (diet and physical activity) to cope with the lockdown compared to participants with just secondary education level. Additionally, closure of university campuses could potentially minimize the access to unhealthy food and drinks options that are commonly available on campus and in the surrounding areas. These findings are in alignment with a study that examined the perceived behavioral changes in a large sample of UK adults during the lockdown, and revealed that lower education level is significantly associated with increased overeating during the lockdown [39]. Our study shows that the lockdown has impacted the Egyptians differently based on their geographical location. Participants from Cairo governorate had higher BMI with 70% of respondents from Cairo were either overweight or obese. This could be attributed to spread of fast food brands, overcrowding, and high cost of living as reported in a recent cross-sectional descriptive study [42]. The geographical location of Alexandria governorate being close to the Mediterranean Sea made respondents more relying on sea food and less on fried food and commercial pastries compared to other geographical regions [33]. It is interesting to find that respondents from Cairo cooked more during the lockdown compared to participants from Alexandria, which could be associated with more healthy food choice.

Similar to a previous study [24], our study has also demonstrated some significant difference in dietary habits between males and females during the lockdown. For example, female Egyptians preferred vegetables, butter, margarine, honey, homemade pastries cooked vegetables, pasta, rice with increasing frequency of cooking. On the other side, male Egyptians consumed higher amounts from carbonated beverages, fish seafood, fast food and increased physical activity during lockdown. It should be noted that the responsibilities of cooking, grocery shopping, and providing home care for children rely mostly on the women. Therefore, it is not surprising to see increased intake of home-made pastries and higher cooking frequency among Egyptian women during the lockdown. On the other hand, men in Egypt are the ones responsible for providing living expenses for their families and therefore they go outside and work. Consequently, Egyptian men reported increased intake of carbonated beverages and fast food and had more weight gain compared to women. These findings were also consistent with the structured questionnaire of Schroder et al. for eating habits and adherence to the Mediterranean Diet pattern during the COVID-19 lockdown [16].

Our study is not devoid of limitations, and therefore, the reported findings should be interpreted with careful consideration of such limitations. Most of the study respondents were from the northern part of Egypt while other regions such as the Canal cities, Upper Egypt, and Sinai were not fairly represented. We acknowledge the cultural differences between the different regions in Egypt, and therefore we expect different findings if we had equal representation from all geographical regions in Egypt. However, our study included the two largest well known governorates of Egypt (Cairo and Alexandria). Considering the cross-sectional design of this study, it is not feasible to establish a direct cause-effect relationship between the lockdown, dietary and personal behaviors, and weight changes. Finally, while the study reported observed changes, we were unable to measure the magnitude of these changes (e.g., % weight increase or decrease).

Although the number of male participants is still remarkable “229” to reflect the dietary habits changes between males and females, we acknowledged this imbalance in gender representation as one limiting factor in our study. It is interesting to find that similar degree of gender imbalance (where female is > 1.5x male participants) is very common in similar studies (most of them are cited in this manuscript). We have mentioned that our future goal is to design further studies and make sure we have equal gender representation through aggressive marketing of the survey and keep collecting data until a certain % of each gender is met.

Further studies covering all regions of Egypt and have similar representation from participants of all genders and educational background are required in the future.

## Conclusion

In conclusion, we provide for the first time an insight of the dietary and physical changes in the adult Egyptian population during the COVID-19 lockdown. From our study, it is evident that age, body weight, educational level, geographical location, and gender play key factors in determining perception of adult Egyptians to the lockdown. Our findings provide essential information for the Egyptian Ministry of health to broadcast recommendations in the future pandemic lockdown about the awareness of healthy dietary habits and physical activity to protect their health from enhance their immune system against infection complications [34].

## Electronic supplementary material

Below is the link to the electronic supplementary material.

Supplementary Material 1

## Data Availability

All data generated or analyzed during this study are included in this published article and its supplementary information file.
