# Supplementary material for: Changes in dietary habits during Covid-19 lockdown in Egypt: the Egyptian COVIDiet study
Source: BMC Public Health. 2023 May 25;23:956. doi: 10.1186/s12889-023-15777-7 (PMC10209922; doi:10.1186/s12889-023-15777-7)
Supplement: Supplementary file 1 — Supplementary Material 1 [file 12889_2023_15777_MOESM1_ESM.pdf]

Table S1: English version of COVIDiet questionnaire

1. **Is olive oil the main source of fat that is added to cooking?**
   a) Yes.
   b) No.

2. **How much olive oil do you consume per day (including frying, meals outdoors, salads, etc.)?**
   a) 1- 0 spoon.
   b) 3- 1 tablespoons.
   c) 4 tablespoons or more.

3. **Do you use more olive oil in eating during confinement than you usual?**
   a) No. Lower.
   b) As usual.
   c) Yes. Higher.

4. **How often do you consume vegetables? (1 serving = 222 g)?**
   a) 1- 0.
   b) 2 -1.
   c) More than 2.

5. **Did you increase your vegetable intake during confinement compared to your usual intake?**
   a) No. Lower.
   b) As usual.
   c) Yes. Higher.

6. **How many pieces of fruit (including fresh juice) do you consume per day?**
   a) 1- 0.
   b) 2- 1.
   c) More than 2.

7. **Did you increase your fruit intake during confinement compared to your usual intake?**
   a) No. Lower.
   b) As usual.
   c) Yes. Higher.

8. **How many servings of red meat, hamburger, sausage, or cold cuts do you consume per day? (Meal: 122- 152 g).**
   a) I don't eat red meat.
   b) One meal.
   c) More than one meal.

9. **Did you increase your intake of red meat, hamburgers, sausage, or cold cuts during confinement compared to your usual intake?**
   a) No. Lower.
   b) As usual.
   c) Yes. Higher.

10. **How many tablespoons of butter, Margarine or cream do you consume each day? (Individual serving: 1 tablespoon):**
    a) 1- 0
    b) More than 1

11. **How many Carbonated or sugary drinks do you consume in a day?**
    a) 1 -0.
    b) More than 1.

12. **Did you increase your intake of carbonated or sugary drinks during the confinement period compared to your usual intake?**
    a) No. Lower.
    b) As usual.
    c) Yes. Higher.

13. **Do you eat honey?**
    a) No.
    b) Yes.

14. **How many times do you consume honey in a week? -**
    a) I never eat honey.
    b) Once a week.
    c) 3- 1 times a week.
    d) Every day.

15. **How many servings of legumes do you consume in a week? (Meal = 1 dish = 152 g):**
    a) 1- 0.
    b) 2 -1.
    c) Three or more times.

16. **Did you increase your intake of legumes during the confinement period compared to your usual intake?**
    a) No. Lower.
    b) As usual.
    c) Yes. Higher.

17. **How many times a week do you eat seafood? 1 piece: 152-122 g of fish or 5- 4 pieces or 222 g of seafood?**
    a) Once or less.
    b) Once or twice.
    c) Three or more times.
    d)

18. **Did you increase your intake of fish and seafood during the con period confinement compared to your usual intake?**
    a) No. Lower.
    b) As usual.
    c) Yes. Higher.

19. **How many times did you eat commercials (not homemade) pastries such as biscuits, sweets, or cakes in a week? -**
    a) Once or less.
    b) Two or more times.

20. **Did you increase the intake of commercial pastries (not made at home) such as biscuits, sweets, or cakes while confinement compared to your usual eating?**
    a) No. Lower.
    b) As usual.
    c) Yes. Higher.

21. **Did you increase the intake of homemade pastries such as biscuits, sweets, or cakes during the confinement period compared to your eating the usual?**
    a) No. Lower.
    b) As usual.
    c) Yes. Higher.

22. **How many times do you eat nuts a week?**
    a) Once or less.
    b) Once or twice.
    c) Three or more times.

23. **Would you prefer chicken, turkey, or rabbit meat instead of beef, hamburger, or sausage?**
    a) No.
    b) Yes.

24. **How many times a week do you consume cooked vegetables, pasta, rice, or other dishes spiced with tomatoes, garlic, onions, or sauce?**
    a) Once or less.
    b) Twice.
    c) Three or more.

25. **How many meals (breakfast, lunch, or dinner) did you eat outside the house before confinement?**
    a) There is no.
    b) 1.
    c) 2.
    d) 3.

26. **What kind of cooking does this mother use?**
    a) Oven.
    b) Frying.
    c) Steamed or boiled.
    d) The microwave.

27. **Do you cook more than before?**
    a) No. Lower.
    b) As usual.
    c) Yes. Higher.

28. **Have you increased your intake of fried foods?**
    a) No. Lower.
    b) As usual.
    c) Yes. Higher.

29. **During the confinement period, how often do you consume fried food?**
    a) Less than once a week.
    b) 3 -1 times a week.
    c) 6 -4 times a week.

30. **When you consume fried foods, what type of oil do you use?**
    a) Sunflower oil.
    b) Olive oil.
    c) Other.

31. **Did you increase the frequency of snacking during the confinement period compared to your usual intake?**
    a) No. Lower.
    b) As usual.
    c) Yes. Higher.

32. **Did you increase your intake of fast food during the confinement period compared to your usual intake?**
    a) No. Lower.
    b) As usual.
    c) Yes. Higher.

33. Do you think you are eating more than usual during the confinement period?

a) No
b) Yes

34. Have you modified your physical activity?

a) I do not do physical activity.
b) Has fallen.
c) As usual.
d) It has increased.

35. **Did you gain weight during the confinement period?**

a) Not dropped.
b) Yes increased.
c) My weight is the same.
